# Synthesis, characterization, and evaluation of the antifungal properties of tissue conditioner incorporated with essential oils-loaded chitosan nanoparticles

**DOI:** 10.1371/journal.pone.0273079

**Published:** 2022-08-19

**Authors:** Hina Ashraf, Hashmat Gul, Bushra Jamil, Asfia Saeed, Mehwish Pasha, Muhammad Kaleem, Abdul Samad Khan

**Affiliations:** 1 Department of Dental Materials, Army Medical College, National University of Medical Sciences, Islamabad, Pakistan; 2 Department of Dental Materials, Ayub Medical College, Abbottabad, Pakistan; 3 Department of Microbiology, BJ Micro Lab, Rawalpindi, Pakistan; 4 Department of Dental Materials, Islamabad Medical & Dental College, Shaheed Zulfiqar Ali Bhutto Medical University, Islamabad, Pakistan; 5 Department of Dental Materials, Shifa College of Dentistry, Shifa Tameer-e-Millat University, Rawalpindi, Pakistan; 6 Department of Restorative Dental Sciences, College of Dentistry, Imam Abdulrahman Bin Faisal University, Dammam, Saudi Arabia; The Islamia University of Bahawalpur Pakistan, PAKISTAN

## Abstract

**Purpose:**

This study aims to investigate new tissue conditioner (TC) formulations involving chitosan nanoparticles (CSNPs) and essential oils (EO) for their antifungal potential, release kinetics, and hardness.

**Materials and methods:**

CSNPs were synthesized, and the separate solutions of CSNP were prepared with two types of EO, i.e., Oregano oil and Lemongrass. The EO was loaded separately in two concentrations (200 μL and 250 μL). The blank and EO-loaded CSNPs were screened against *Candida albicans* (*C*. *albicans*), and their minimum inhibitory concentration was established. GC Reline^™^ (GC corporation, USA) TC was considered a control group, whereby the four experimental groups were prepared by mixing CSNPs/EO solutions with TC powder. The antifungal effectiveness (*C*. *albicans*) and release kinetics behavior (1–6 h, 24 h, 48 h, and 72 h) was investigated. The Shore A hardness of control and experimental groups was evaluated in dry and wet modes (deionized water and artificial saliva). For statistical analysis, SPSS version 22 was used to do a one-way ANOVA post-hoc Tukey’s test.

**Results:**

Compared to the control group, TCs containing blank CSNPs and CSNPs loaded with EO showed 3 and 5 log reductions in *C*. *albicans* growth, respectively. A significantly high antifungal effect was observed with TC containing lemongrass essential oil (200 μL). The continuous release of EO was detected for the first 6 hours, whereas completely stopped after 48 hours. Mean hardness values were highest for dry samples and lowest for samples stored in artificial saliva. The statistically significant difference within and between the study groups was observed in mean and cumulative essential oils release and hardness values of TCs over observed time intervals irrespective of storage media.

**Conclusion:**

TCs containing essential-oil-loaded CSNPs seem a promising alternative treatment of denture-induced stomatitis, however, a further biological analysis should be taken.

## Introduction

Tissue conditioners are temporary soft denture liners that improve the adaptation of removable dentures and act as a cushioning material. The tissue conditioners help to redistribute the occlusal and masticatory stresses placed on underlying oral mucosa [1]. Nonetheless, tissue conditioners have limitations, including the loss of resilience over time, dimensional changes, and microbial colonization [1, 2].

Approximately 70% of the denture wearers experience inflammation of the denture base mucosa i.e., “denture stomatitis”. It is often caused by the colonization of dentures by *Candida albicans (C*. *albicans)* and is more frequently reported among women and older people [3–6]. Different therapeutic options are available for treating denture stomatitis, including replacing ill-fitting dentures, improving oral and denture hygiene, using local or systemic antifungal medications, and using tissue conditioners [7–9]. However, the microporous nature of tissue conditioners and their tendency to form micro-porosities by leaching constituents over time may contribute to microbial colonization, which can further aggravate denture stomatitis [10, 11].

Various antimicrobial agents, including metal oxides, photocatalysts, silver nanoparticles, zeolite, chlorhexidine, fluconazole, nystatin, miconazole, clotrimazole and chitosan have been added to tissue conditioners to maintain adequate denture hygiene and minimize their potential for microbial colonization [12–14]. In recent years, the use of natural antimicrobial agents such as chitosan and herbal extracts has been explored [15]. Chitosan, a natural biopolymer [16], is a non-toxic, biocompatible, biodegradable material with superior antimicrobial properties [17], and can act as a drug carrier [18]. The antibacterial and antifungal potential of tissue conditioner containing chitosan nanoparticles has been reported previously [14, 19].

The incorporation of essential oils such as *Carum copticum L*. into tissue conditioner has been investigated. It was observed that thymol (a component of essential oil) has broad antimicrobial activity [20]. In another study, the essential oil of *Melaleuca alternifolia* (tea tree oil) was added to a tissue conditioner and revealed a potent antifungal effect [21]. Oregano oil *(origanum vulgare)* that contains two basic components, carvacol and thymol, whereas Lemongrass oil *(Cymbopogan citratus*) containing a major component; citral, have been incorporated into tissue conditioner. Both of them showed significant antibacterial and antifungal activity [22, 23]. However, these essential oils are volatile plant extracts with a limited antibacterial effect.

The use of chitosan nanoparticles as a drug delivery vehicle for essential oils was aimed at providing a synergistic antifungal effect. Moreover, allowing the controlled release of essential oils, making them more effective against denture stomatitis [24, 25]. Chitosan nanoparticles possess favorable properties such as biocompatibility, ability to bind with organic compounds, biodegradability, inherent physiological activity, and mucoadhesive nature. These properties have been exploited by researchers to explore their potential as a drug delivery molecule [26]. Chitosan, because of its reactive nature, can be produced in various dispensing formulations such as powder, paste, film, fiber, gel, etc. Chitosan has been chemically modified to produce a variety of derivatives (carboxylated, conjugated, thiolated, and acylated chitosan derivatives) to overcome the burst release of loaded drugs and make it a sustained-release system. These modified formulations of chitosan are used in various drug delivery systems [26].

Therefore, the objectives of this study were two-fold, initially, to synthesize and characterize chitosan nanoparticles (CSNPs), both blank and loaded with essential oils. Furthermore, incorporate these nanoparticles into tissue conditioner to evaluate their antifungal potential against *C*. *albicans*, release kinetics, and hardness of modified tissue conditioner upon ageing. It was hypothesized that the tissue conditioners with CSNPs-loaded essential oils would inhibit *Candida* growth via sustained essential oil release and provide a synergistic antimicrobial effect.

## Materials and methods

Chitosan powder, glacial acetic acid (CHEBI 15366; purity>99.7%), and sodium tripolyphosphate solution were purchased from Sigma Aldrich, St. Louis, MO, USA. Essential oils of Oregano (H.S. code#33242387) and Lemongrass (H.S. code#33012942) were obtained from the National Agriculture Research Centre, Rawalpindi, Pakistan.

### Preparation of blank chitosan nanoparticles

Blank CSNPs were prepared by adding 1 mL of a 1% sodium tripolyphosphate solution dropwise to a chitosan solution under constant stirring over 3–4 hours. The blank nanoparticles were designated as BNP. Prior to any testing, the CSNP-containing solution was stirred with a magnetic stirrer (Corning PC-420D, Seoul, South Korea) for 15 min at room temperature to ensure the homogenous dispersion of nanoparticles. The drops of nanoparticles spread uniformly on the glass slide and were left to dry [27]. The dried nanoparticles were inspected with a scanning electron microscope (JEOL JSM-6390 LA, Tokyo, Japan) at a resolution of 10kV and 5,000–50,000 x.

### Synthesis of chitosan nanoparticles loaded with essential oils

CSNPs loaded with essential oils (EOs) were made by ionic gelation method with a few modifications [26]. Briefly, 0.075g of chitosan powder was added to a 1% (w/w) acetic acid solution under constant agitation, which continued for 30 min at room temperature. For active loading of EOs on the CSNPs, 200μL and/or 250μL of Oregano essential oil was dissolved separately in 1mL of a 1% sodium tripolyphosphate (TPP) solution. Then both solutions were added separately drop by drop to 12.5mL of chitosan solution under constant agitation over 4 hours. This was followed by ultrasonication (UP400S Hielscher Ultrasonics, GmbH, Frankfurt, Germany) for 15 min at 37°C to ensure homogenous dispersion of nanoparticles. This yielded 12.5mL of CSNPs loaded with Oregano essential oil in distilled water, designated as ONP1 (with 200 μL of essential oil) and ONP2 (with 250μL of essential oil). The same procedure was repeated with Lemongrass essential oil to make CSNPs loaded with Lemongrass essential oil, designated as LNP1 (with 200μL of essential oil) and LNP2 (with 250μL of essential oil). These CSNPs loaded with essential oils were then observed with the scanning electron microscope.

### Preparation of experimental samples

GC Reline tissue conditioner, GC Reline^™^ (GC corporation, USA) was used as the control group. Following the manufacturer’s instructions, it was mixed according to the powder-to-liquid ratio of 2.2g to 1.8mL. To prepare experimental tissue conditioner, part of the liquid portion of the tissue conditioner was replaced with the solution of CSNPs loaded with essential oils. The liquid was then mixed with the powder portion. The liquid portion of tissue conditioner was incorporated into the experimental solution at a ratio of 4:1 with repeated pipetting to ensure homogenous mixing of the tissue conditioner liquid and the experimental solution. Then the powder portion of tissue conditioner was incorporated into the liquid by mixing with a sterile spatula for 30s. The mix was then poured into custom-made prefabricated round Teflon molds (10×1 mm^2^) and rectangular stainless-steel molds (100-mm×20-mm×10-mm) [27]. The yielded tissue conditioner discs loaded with essential oils and CSNPs were in a semi-solid form. The tissue conditioner discs were grouped according to their formulation, as shown in Table 1.

**Table 1:** Groups of tissue conditioners used in the experiment (n = 3).

Sr. No.	Group Name	Composition
**1**.	ECO-200 (Experimental TC)	PEMA (poly-ethyl methacrylate-based tissue conditioner) + ONP1 (200μL Oregano oil loaded CSNPs)
**2**.	ECO-250 (Experimental TC)	PEMA+ ONP2 (250μL Oregano oil loaded CSNPs)
**3**.	ECL-200 (Experimental TC)	PEMA+ LNP1 (200μL Lemongrass oil loaded CSNPs)
**4**.	ECL-250 (Experimental TC)	PEMA+ LNP2 (250μL Lemongrass oil loaded CSNPs)
**5**.	ECB (Experimental TC)	PEMA+ BNP (Blank CSNPs)
**6**.	CTC (Control TC)	PEMA

### Antifungal analysis

An agar well diffusion assay was carried out to measure the zones of inhibition formed by essential oils against *C*. *albicans* (ATCC, 90029), following the method described previously [28, 29]. The minimum inhibitory concentration (MIC) of CSNPs loaded essential oils solution was determined using the broth micro-dilution method, as per ISO 16256:2012 specifications and NCCLS (National Committee for Clinical Laboratory Standards) [30]. All experiments were performed in triplicate. MIC was recorded by visually inspecting changes in turbidity of the experimental wells and by comparing them with the positive and negative controls. Turbidity was classified with numbers between 0 and 4, according to Clinical and Laboratory Standards Institute guidelines [29].

The agar disc diffusion method determined the antifungal effect of control and experimental tissue conditioner discs (10×1 mm^2^) on *C*. *albicans* [31, 32]. Colony-forming units (CFU) were counted and converted to CFU/mL using the following formula:

$$CFU/mL=\frac{Number\;of\;colony\;forming\;units\;\left(CFU\right)}{Amount\;plated\;\left(mL\right)\times Dilution\;factor}$$ (1)

### Release kinetics

Release characteristics of the control and experimental tissue conditioner discs (n = 3) were analyzed with a UV/Vis spectrophotometer (Bruker ALPHA Platinum-ATR spectrometer, Bremen, Germany). The disc samples (10×1 mm^2^) were prepared as described earlier and placed in a drying oven (ESCO forced convection oven, OFA 54–8 Singapore) at 37°C for 1 hour. Samples were stored in 3mL of distilled water. During the first 6 hours, 1mL of the sample solution was removed at each 1-hour interval and replaced with fresh distilled water. Then the solution that had been removed was analyzed with UV/Vis spectrophotometer set at a 257–300 nm wavelength range. The same procedure was repeated after 24 hours, 48 hours, and 72 hours for all sample discs [22]. The experiment was performed in triplicate.

### Hardness testing

A total of 54 tissue conditioner blocks (100mm × 20mm × 10mm) were used. Three samples were used for each of the following conditions: dry, moistened with distilled water, and moistened with artificial saliva (n = 3). The artificial saliva was prepared as described in literature [33]. A digital Shore A durometer (Novotest TS-C, Novomoskovsk, Ukraine) was used to carry out hardness testing for tissue conditioner groups (control and experimental), following the procedure from the American Society for Testing and Materials (ASTM) 2240–05 (2010), with a dwell time of 5 seconds. The first reading was taken immediately after sample gelation (1 hour). After this, samples (n = 3) were stored in either artificial saliva or distilled water or remained dry and wrapped in aluminum foil at 37°C. Hardness readings (6 readings per sample, 6mm apart from each other and 10mm away from the edges) were taken at regular time intervals on days 1, 3, and 5.

### Statistical analysis

For data analysis, the Statistical Package for Social Sciences (SPSS) software version 22 was used (IBM Software, Armonk, NY, USA). For categorical variables, frequencies and percentages were calculated. Means and standard deviations were calculated for numerical variables. For comparative variables, ANOVA and post-hoc Tukey’s test were done to calculate the statistical difference between and within groups. Values of *p*<0.05 were considered significant.

## Results

**Fig 1:**
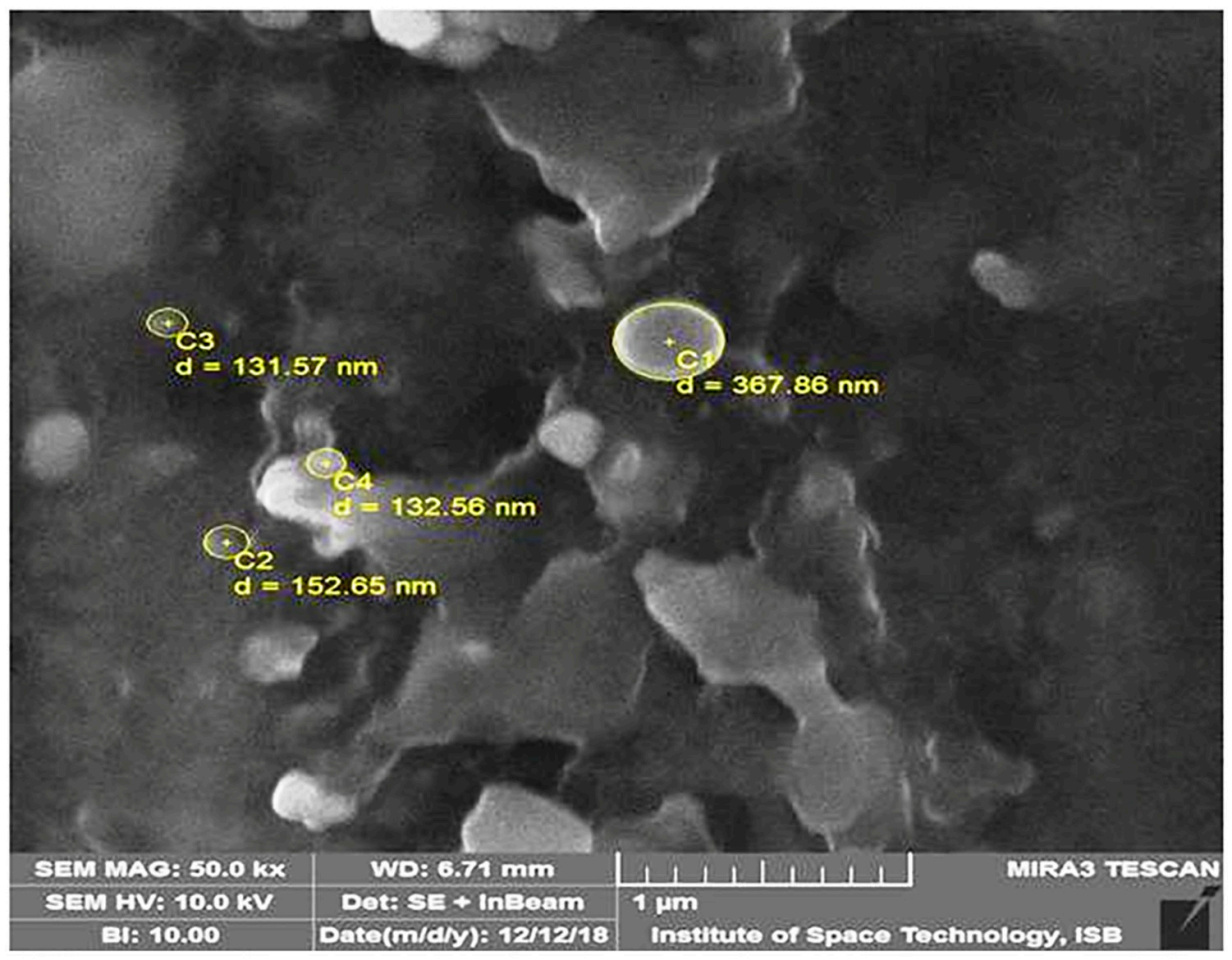
Morphological evaluation of chitosan nanoparticles loaded with oregano oil by scanning electron microscopy at 50000 x and 10.0 kV. The nanoparticles are in the range of 40–500 nm and show some agglomeration.

The prepared CSNPs loaded with essential oils were spherical in shape, with a size of 100–200 nm, and with some aggregation as shown in Fig 1.

**Fig 2:**
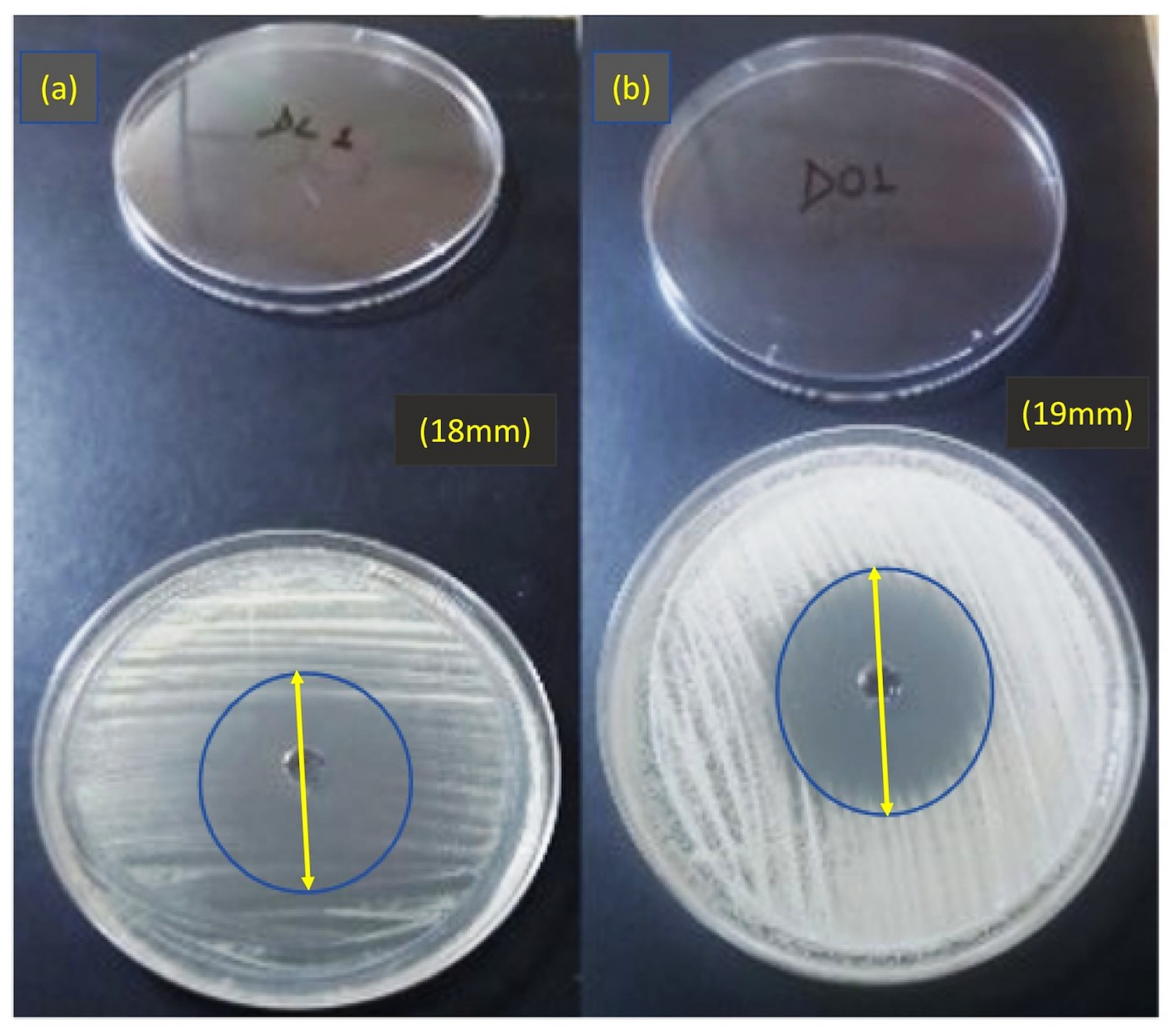
Essential oils screening; Zone of inhibition of chitosan nanoparticles loaded with (a) Lemongrass oil (L) and (b) Oregano oil (O), where the appeared zone of inhibition was 18 mm and 19 mm, respectively.

Upon screening in the agar well diffusion assay, both Oregano and Lemongrass essential oil had prominent zones of inhibition against *C*. *albicans*, as shown in Fig 2.

The MIC of Oregano oil was 200±0.007 μL/mL, and that of Lemongrass oil was 156±0.006 μL/mL.

### Antifungal analysis

**Fig 3:**
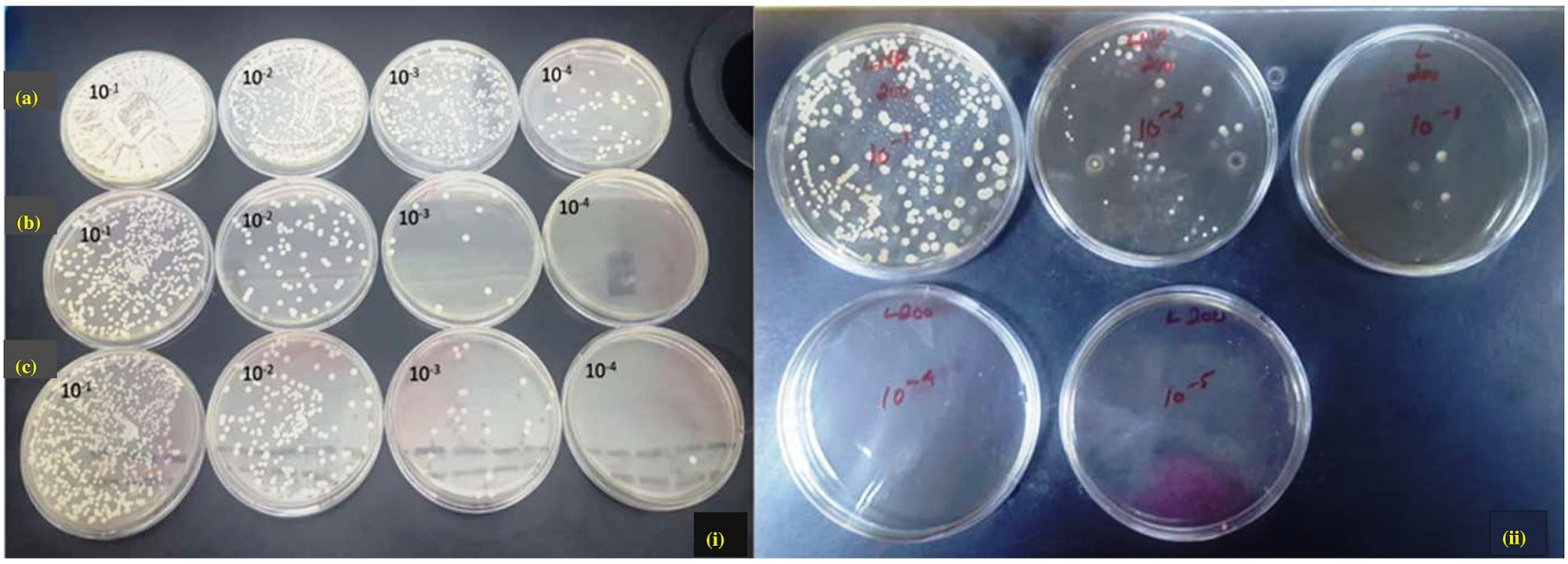
CFU counts of C. *albicans* at different dilutions; (i) (a). tissue conditioner discs without chitosan (control), (b). tissue conditioner discs with ECO 200, (c). tissue conditioner discs with ECO 250 and (ii) tissue conditioner discs with ECL 200, control TC disks were easily colonized by *C*.*albicans* till the last dilution whereas experimental TC disks hindered the growth of *C*.*albicans* rapidly. At the 10^−3^ dilution, there was negligible growth on the plate for ECO 200, ECO 250, and ECL 200.

The antifungal effect of control and experimental tissue conditioner discs was evaluated based on the density of *C*. *albicans* attached to these groups. The CFU results are given in Fig 3i & 3ii.

**Fig 4:**
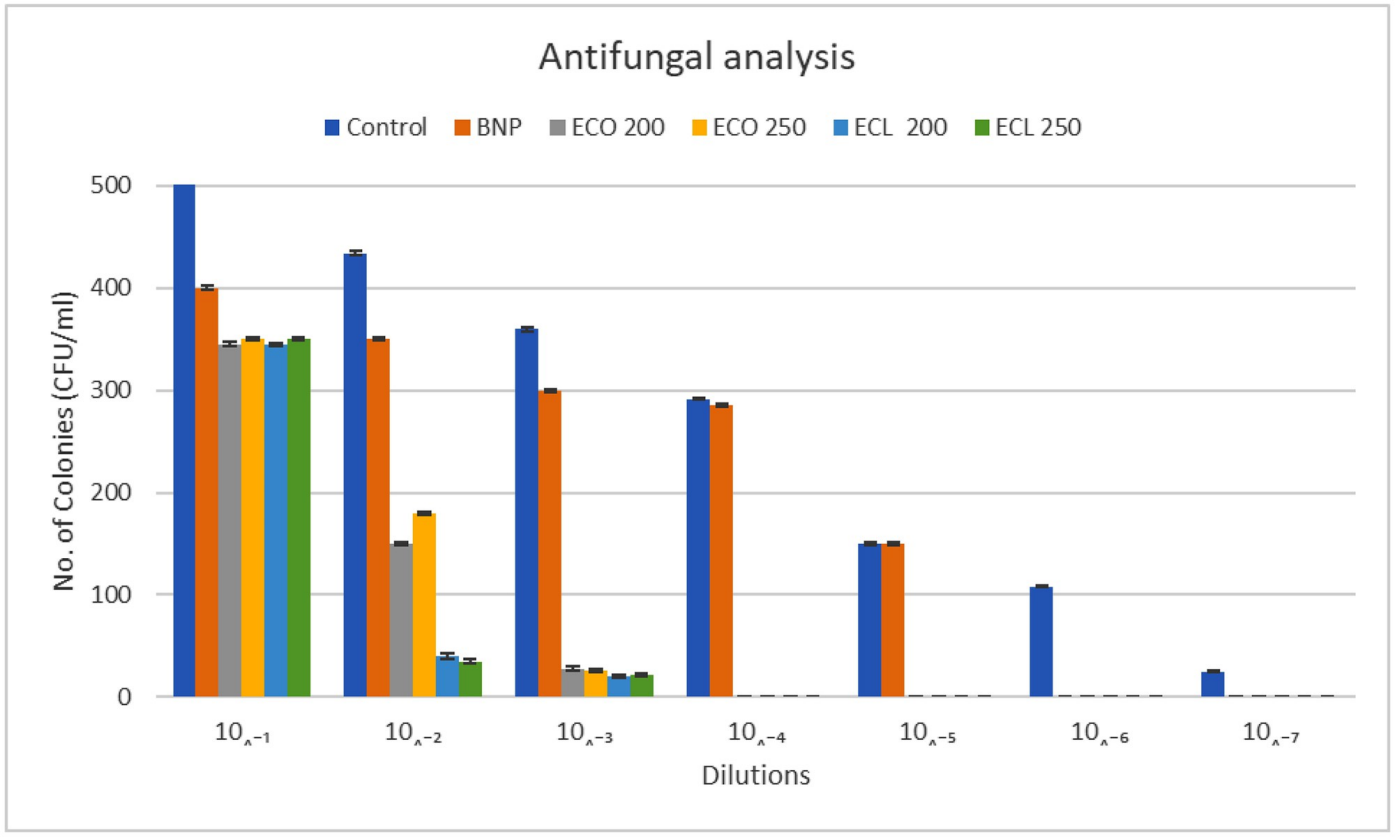
CFU counts of C. *albicans* on the surface of control tissue conditioner, blank chitosan nanoparticles, and chitosan nanoparticles loaded with essential oils (in both concentrations) based tissue conditioners. The graph depicts that the experimental groups show significantly low CFU/mL compared to the control and the blank chitosan nanoparticles.

It was observed that control tissue conditioner discs were easily colonized by *C*. *albicans* until the 10^−8^ dilution, while the experimental tissue conditioner discs quickly hindered the growth of *C*. *albicans*. At the 10^−3^ dilution, there was negligible *Candida* growth on the plate for all EO-loaded tissue conditioner groups (ECO 200, ECO 250, ECL 200, and ECL 250). The *Candida* growth on tissue conditioner groups loaded with essential oils decreased to zero on the 10^−4^ dilution, as shown in Fig 4.

There was a 3-log reduction of *Candida* growth in BNPs compared to the controls. For the other four groups (ECO 200, ECO 250, ECL 200, and ECL 250), there was 5-log reductions in *Candida* growth compared to the control, as shown in Table 2.

**Table 2:** Growth of *C*. *albicans* on control (CTC) and experimental (BNP, ECO 200, ECO 250, ECL 200, ECL 250) TC discs.

Groups	10^−1^	10^−2^	10^−3^	10^−4^	10^−5^	10^−6^	10^−7^
CTC	Ӿ	Ӿ	Ӿ	Ӿ	Ӿ	Ӿ	Ӿ
BNP	Ӿ	Ӿ	Ӿ	Ӿ	__	__	__
ECO 200	Ӿ	Ӿ	__	__	__	__	__
ECO 250	Ӿ	Ӿ	__	__	__	__	__
ECL 200	Ӿ	Ӿ	__	__	__	__	__
ECL 250	Ӿ	Ӿ	__	__	__	__	__

*(Ӿ) shows positive growth, (-) shows no growth.

One-way ANOVA showed statistically significant differences in *Candida* colonization of all the tissue conditioner disc groups (*p*<0.01). Pairwise comparison showed a statistically significant difference in *Candida* colonization between all tissue conditioner groups, except between ECO 200 and ECL 200 (*p* = 0.996) and between ECO 250 and ECL 250 (*p* = 1.00). These findings were confirmed by CFU counts at different dilutions of experimental tissue conditioner discs, as shown in Figs 3 and 4.

### Release kinetics

**Fig 5:**
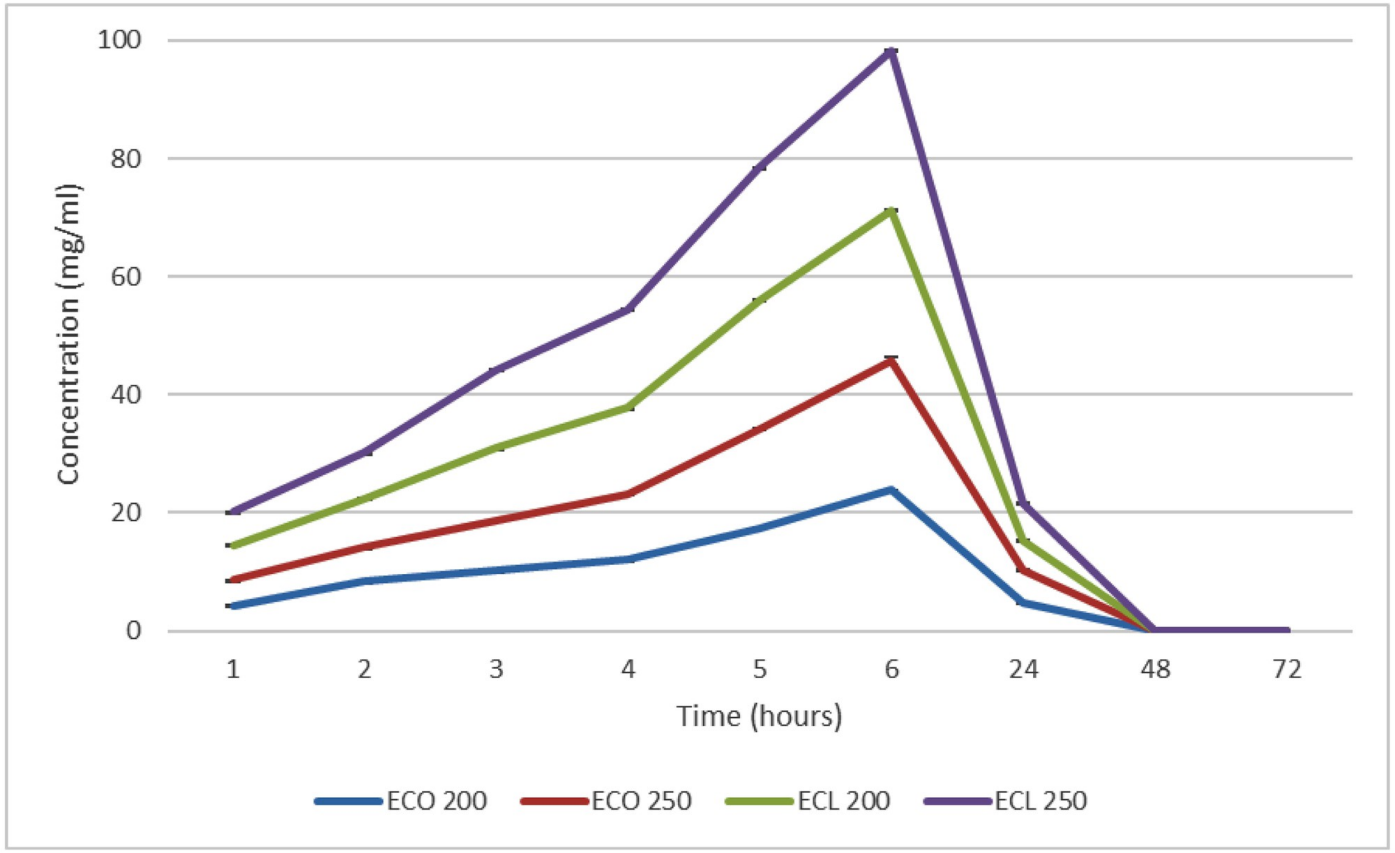
Concentration of EOs release from tissue conditioner discs (ECO 200, ECO 250, ECL 200, ECL 250) over 1 h, 2 h, 3 h, 4 h, 5 h, 6 h, 24 h, 48 h, and 72 h. Values were expressed as means and standard deviation. The maximum release was observed at 6 h, the release pattern was reduced. Highest EO release was observed by ECL 250, followed by ECL 200, ECO 250 and least was for ECO 200.

The oils showed a continuous gradual release for the first 6 hours on day 1. There was no release on the second and third days, as shown in Fig 5.

A statistically significant difference (*p*<0.01) was observed in mean and cumulative EO release values within each study group over the observed time intervals. A statistically significant difference in mean hourly EO release and cumulative EO release was noted between the four EO containing experimental study groups (*p*<0.01).

### Hardness testing

**Fig 6:**
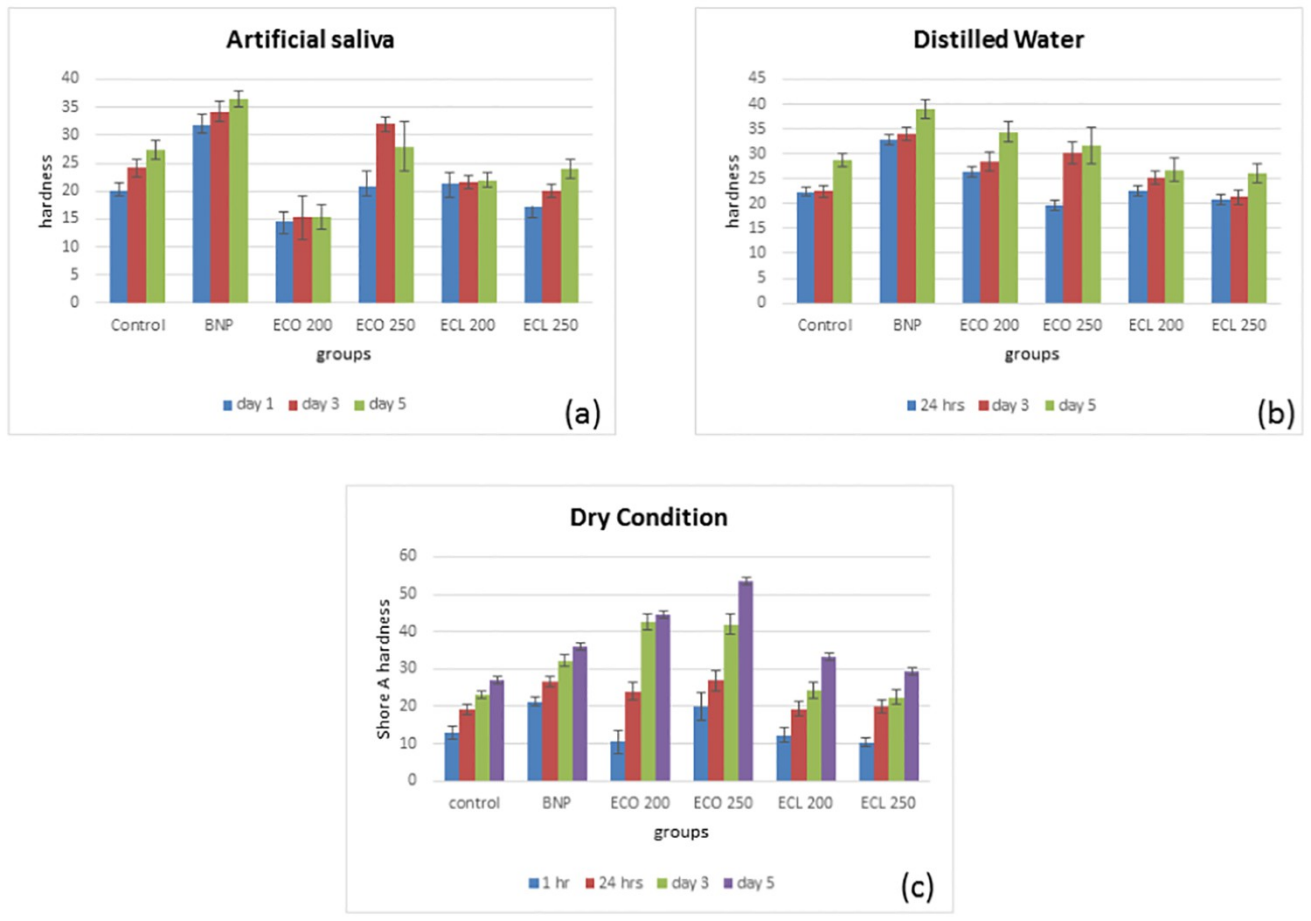
Mean Shore A hardness of control and experimental TC discs over 5 days in (a) artificial saliva, (b) distilled water and (c) dry conditions. It was found that the hardness values were high compared to the wet mode, whereby a non-significant difference was found between the distilled water and artificial saliva.

Irrespective of the storage medium, the experimental and control groups displayed a statistically significant rise in hardness values over the designated time (*p<*0.05). The mean hardness values were highest for dry samples and lowest for samples stored in artificial saliva. This was true for both control and experimental groups. A statistically significant difference (*p*<0.01) was observed in hardness values between aged samples stored in different media, both within and between the control, and the experimental groups of tissue conditioner discs: dry condition, *p* = 0.009; distilled water, *p* = 0.008; and artificial saliva, *p* = 0.0087. The Shore A hardness values of control and experimental groups of tissue conditioner samples are presented in Fig 6a–6c.

## Discussion

Tissue conditioners are temporary soft denture liners that are placed between the oral mucosa and denture-bearing area. They act as a cushioning material to redistribute occlusal and masticatory stresses on the oral mucosa and eventually help condition hyperemic and sore mucosa [34]. They can also act as a drug delivery vehicle for managing denture stomatitis [35]. Chitosan has several modifiable functional groups that aid in its application as a drug delivery system [36]. In this study, CSNPs were used as a drug delivery vehicle for essential oils in tissue conditioner to synergize the antimicrobial effect of oils and to ensure their controlled release. This study shows that the addition of CSNPs to tissue conditioner generates an antifungal effect in-vitro and that this effect is amplified when CSNPs loaded with essential oils are added to tissue conditioner. CSNPs loaded with essential oils have great potential as a cure for candidal denture stomatitis.

The size range of the CSNPs loaded with essential oils fell within the acceptable range established in previous studies, which reported similar characteristics in CSNPs with sizes varying from 40–500 nm [28, 32, 37]. The increase in the size of the CSNPs loaded oil can be ascribed to the presence of oil in them [38]. The uniform distribution of CSNPs seems to be beneficial, as it may help their even distribution within the tissue conditioner following mixing. The size, stability, and surface potential of CSNPs directly affect their ability to encapsulate and release drugs [39].

Notably, in aqueous environments, CSNPs tend to absorb water, resulting in greater swelling, followed by a burst release of loaded molecules. This is a potential limitation to their use [34, 35]. To overcome the problem of burst release of loaded drugs and make a sustained release system, chitosan has been chemically modified to produce a variety of derivatives. These are carboxylated, conjugated, thiolated, and acylated chitosan derivatives. These modified formulations of chitosan are used in various drug delivery systems [40].

In the present study, no inhibition of *C*. *albicans* was seen in the control group (CTC) or BNP. However, the other four experimental groups (ECO 200, ECO 250, ECL 200, and ECL 250) showed a significant reduction in candidal growth. Similar results have been reported in previous studies, which demonstrated that tissue conditioners alone could not hinder the growth of *C*. *albicans*, however, the incorporation of antifungal agents into tissue conditioners could inhibit fungal colonization [21]. The greatest reduction in *Candida* growth was observed for CSNPs loaded with Lemongrass oil (ECL 200 and ECL 250). A previous study [13] had assessed the fungicidal and fungistatic effects of different essential oils against *C*. *albicans*, and had reported that citral, a major component of lemongrass oil, demonstrated the best antifungal response. The breakdown of lipids in bacterial and fungal cell membranes is due to the hydrophobic nature of essential oils. This causes the components of cells to leak, eventually causing cell death [38]. The antifungal response exhibited by CSNPs loaded with Oregano oil is possibly due to the presence of carvacol, thymol, and terpenoids in oregano oil [23, 28].

In this study, chitosan and essential oils had a synergistic antifungal effect against *Candida* colonization. Chitosan has antimicrobial properties due to its polycationic structure, which allows it to interact with anionic bacterial cell membranes. This increases the permeability of cell membranes, causing significant membrane depolarization [15]. However, only a few studies are available on the use of combined chitosan and essential oils with tissue conditioner for the treatment of denture stomatitis [14, 41]. In this current study, the release of essential oils from tissue conditioner samples increased over the first six hours for all the groups, reaching its maximum at six hours. After this, it declined until the end of day 1, with no more release on day 2 or 3. The release of essential oils from CSNPs can be attributed to a biphasic process, consisting of an initial fast release during the first phase for six hours and a slower release during the second phase until 24 hours elapsed. The initial release is likely due to the oil molecules adsorbed onto the surface of the nanoparticles [42]. Essential oils are miscible in alcohol, therefore, they are likely to be released along with alcohol from tissue conditioners during first six hours [43]. The higher rate of dissolution of polymer near the surface may have also led to a faster initial release of essential oils near the surface. The second stage of release was marked by the slow release of essential oils until the end of day 1, followed by no further release. The diffusion of oil from the nanoparticles leads to a slow release at this phase. Furthermore, plasticizers and alcohol leach out from tissue conditioners [3], which also contributes to slow release at this point [44]. It has been reported that drug release by diffusion involves the penetration of the medium into the nanoparticles, causing matrix swelling. The glassy polymer is converted into a rubbery matrix; eventually, the drug is diffused out of this swollen rubbery matrix [22]. The final stage is release on days 2 and 3, which is marked by a plateau indicating zero release of EOs. In the current study, high molecular weight chitosan was employed, which could be the cause of the greater release of oils. The nanometric gauge of the CSNPs loaded with essential oils, along with their even distribution within the tissue conditioner, meant that the CSNPs had a greater interactive surface area in the conditioning media, which may have also contributed to greater essential oil release.

Acrylic resin denture liners that contain plasticizers are unstable in an aqueous environment, and plasticizers tend to leach out, causing the material to harden [45]. In case of nanoparticles, the greater surface area can cause more rapid leaching of plasticizers and, ultimately, a faster loss of compliance [37, 46]. The addition of antimicrobial agents to tissue conditioner is thought to impair the plasticizers and penetrate the polymer chain, forming a softened gel. These materials may also increase the water sorption of the tissue conditioner, eventually affecting its hardness. Despite the increase in Shore A hardness values of the experimental tissue conditioners upon aging in different conditions for five days, the hardness values remained within clinically acceptable range. A range of 13 to 49 Shore A units in 24 hours is considered satisfactory [47].

After aging, all tissue conditioner groups containing CSNPs loaded with essential oils exhibited the greatest hardness if stored under dry conditions than if stored in distilled water and had the least hardness when stored in artificial saliva. However, their hardness values varied depending on the type and loading of essential oil in the tissue conditioner. All groups containing CSNPs loaded with essential oils reported the lowest hardness values at all aging intervals when artificial saliva was used as the conditioning medium. This has great clinical significance, as this experimental setup simulates the oral environment. The increase in hardness values observed for the groups after the addition of antimicrobials is not sufficient to interfere with their clinical use. For all groups, the incorporation of oils at concentrations studied in this research should not produce substantial changes within five days.

It is anticipated that the essential oils can be loaded with chitosan nanoparticles at a large scale. Both essential oils and chitosan can be prepared with a natural source; therefore, it is expected that the product would be cost-effective. The proposed methodology in this study should be clinically acceptable. The clinicians have to mix tissue conditioner powder with essential oil/chitosan solution at a chairside, which will be readily available for the patients. This study explains the synergistic antimicrobial effect of chitosan nanoparticles and essential oils on tissue conditioners without significantly disrupting their hardness. However, more research needs to be done on other essential oils and different brands of tissue conditioners. The impact of CSNPs loaded with essential oils on the tissue conditioners’ water sorption and solubility, creep, and gelation time remains to be investigated. To apply this research at large scale, cytotoxicity testing and clinical trials need to be performed. A novel combination of chitosan nanoparticles, essential oils, and tissue conditioners will be a promising alternative for treating and preventing denture stomatitis.

## Conclusion

Chitosan nanoparticles provide an ideal drug delivery system for essential oils because of their better size, surface area, and stability. Tissue conditioner loaded with BNPs exhibited significantly better antifungal properties than chitosan-free tissue conditioner. Furthermore, tissue conditioners containing CSNPs loaded with essential oils showed better antifungal potential than BNPs. The strongest antifungal action was observed from the tissue conditioner enriched with CSNPs loaded with lemongrass essential oil. The tissue conditioner group containing blank CSNPs had higher hardness than the other tissue conditioner groups. The release rate of essential oils for all four experimental groups (ECO 200, ECO 250, ECL 200, and ECL 250) increased continuously for the first six hours and then declined until 24 hours had elapsed. It became negligible on days 2 and 3. As per findings of this study, it is concluded that the combination of chitosan nanoparticles and lemongrass oil is very promising for treating denture stomatitis, however, further studies are required.

## Supporting information

S1 File (DOCX)

S1 Data (XLSX)

S2 Data (XLSX)

S3 Data (XLSX)

S4 Data (XLSX)

S5 Data (XLSX)
