# Supplementary material for: Synthesis, characterization, and evaluation of the antifungal properties of tissue conditioner incorporated with essential oils-loaded chitosan nanoparticles
Source: PLoS One. 2022 Aug 19;17(8):e0273079. doi: 10.1371/journal.pone.0273079 (PMC9390928; doi:10.1371/journal.pone.0273079)
Supplement: S1 File — (DOCX) [file pone.0273079.s001.docx]

Shore A hardness of control and experimental tissue conditioner disks in dry condition

| No. | Time | Mean ± Standard deviation of hardness in dry condition (n=3) | | | | | |
| --- | --- | --- | --- | --- | --- | --- | --- |
|  |  | CTC | BNP | ECO 200 | ECO 250 | ECL 200 | ECL250 |
| 1. | 1 hour | 12.83 ± 1.83 | 21.22 ± 1.10 | 10.41 ± 2.97 | 20.01 ± 1.12 | 12.3 ± 1.79 | 10.19 ± 1.16 |
| 2. | Day 1 | 19.16 ± 1.20 | 26.6 ± 1.25 | 24.02 ± 2.40 | 26.91 ± 2.77 | 19.3 ± 1.97 | 19.97 ± 1.83 |
| 3. | Day 3 | 23.11 ± 1.11 | 32.22 ± 1.58 | 42.75 ± 2.16 | 41.97 ± 2.83 | 24.11 ± 2.12 | 22.44 ± 1.97 |
| 4. | Day 5 | 26.97 ± 1.75 | 35.91 ± 1.75 | 44.52 ± 1.75 | 53.5 ± 1.75 | 33.12 ± 1.75 | 29.47 ± 1.75 |

Shore A hardness of control and experimental tissue conditioner disks in artificial saliva

| No. | Time | Mean ± Standard deviation of hardness in artificial saliva (n=3) | | | | | |
| --- | --- | --- | --- | --- | --- | --- | --- |
|  |  | CTC | BNP | ECO 200 | ECO 250 | ECL 200 | ECL250 |
| 1. | Day 1 | 20.10 ± 1.03 | 31.83 ± 1.36 | 14.5 ± 2.02 | 20.94 ± 1.72 | 21.36 ± 2.10 | 17.23 ± 2.00 |
| 2. | Day 3 | 24.12 ± 1.51 | 34.19 ± 1.75 | 15.2 ± 3.82 | 31.9 ± 1.30 | 21.61 ± 1.18 | 20.91 ± 1.12 |
| 3. | Day 5 | 27.13 ± 1.62 | 36.47 ± 1.34 | 15.3 ± 2.6 | 28 ± 1.46 | 21.97 ± 1.33 | 23.98 ± 1.61 |

Shore A hardness of control and experimental tissue conditioner disks in distilled water

| No. | Time | Mean ± Standard deviation of hardness in distilled water (n=3) | | | | | |
| --- | --- | --- | --- | --- | --- | --- | --- |
|  |  | CTC | BNP | ECO 200 | ECO 250 | ECL 200 | ECL250 |
| 1. | Day 1 | 22.41 ± 0.91 | 32.71 ± 1.18 | 26.35 ± 2.46 | 19.55 ± 1.80 | 22.55 ± 1.58 | 20.83 ± 1.42 |
| 2. | Day 3 | 22.5 ± 1.17 | 33.94 ± 1.37 | 28.36 ± 1.89 | 30.12 ± 2.31 | 25.25 ± 1.42 | 21.21 ± 1.42 |
| 3. | Day 5 | 28.6 ± 1.32 | 38.9 ± 1.90 | 38.3 ± 2.01 | 31.65 ± 2.16 | 26.75 ± 1.31 | 26.11 ± 1.99 |

| **Antifungal analysis**  **Tests of Between-Subjects Effects** | | | | | | | | |
| --- | --- | --- | --- | --- | --- | --- | --- | --- |
| Dependent Variable: Colony Forming Units | | | | | | | | |
| Source | Type III Sum of Squares | df | Mean Square | F | Sig. | Partial Eta Squared | Noncent. Parameter | Observed Power^b^ |
| Corrected Model | 1894487392857.142^a^ | 41 | 46207009581.882 | 521.657 | .000 | .998 | 21387.936 | 1.000 |
| Intercept | 852042857142.842 | 1 | 852042857142.842 | 9619.192 | .000 | .996 | 9619.192 | 1.000 |
| Groups | 352932892857.143 | 5 | 70586578571.429 | 796.892 | .000 | .990 | 3984.458 | 1.000 |
| Dilution_Factor | 1275576434523.808 | 6 | 212596072420.635 | 2400.117 | .000 | .997 | 14400.702 | 1.000 |
| Groups * Dilution_Factor | 265978065476.191 | 30 | 8865935515.873 | 100.093 | .000 | .986 | 3002.776 | 1.000 |
| Error | 3720250000.000 | 42 | 88577380.952 |  |  |  |  |  |
| Total | 2750250500000.000 | 84 |  |  |  |  |  |  |
| Corrected Total | 1898207642857.142 | 83 |  |  |  |  |  |  |
| a. R Squared = .998 (Adjusted R Squared = .996) | | | | | | | | |
| b. Computed using alpha = .05 | | | | | | | | |

**Hardness Tests of within subjects**

| Source | | Type III Sum of Squares | Df | Mean Square | F | Sig. | Noncent. Parameter | Observed Power^a^ |
| --- | --- | --- | --- | --- | --- | --- | --- | --- |
| Hardness_Dry | Sphericity Assumed | 31941.409 | 3 | 10647.136 | 2324.663 | .000 | 6973.990 | 1.000 |
|  | Greenhouse-Geisser | 31941.409 | 2.754 | 11598.614 | 2324.663 | .000 | 6401.888 | 1.000 |
|  | Huynh-Feldt | 31941.409 | 2.977 | 10730.650 | 2324.663 | .000 | 6919.713 | 1.000 |
|  | Lower-bound | 31941.409 | 1.000 | 31941.409 | 2324.663 | .000 | 2324.663 | 1.000 |
| Hardness_Dry * Groups | Sphericity Assumed | 6364.650 | 15 | 424.310 | 92.643 | .000 | 1389.638 | 1.000 |
|  | Greenhouse-Geisser | 6364.650 | 13.769 | 462.228 | 92.643 | .000 | 1275.641 | 1.000 |
|  | Huynh-Feldt | 6364.650 | 14.883 | 427.638 | 92.643 | .000 | 1378.823 | 1.000 |
|  | Lower-bound | 6364.650 | 5.000 | 1272.930 | 92.643 | .000 | 463.213 | 1.000 |
| a. Computed using alpha = .05 | | | | | | | | |

|  |
| --- |
|  |
|  |
